# Supplementary material for: A Systematic Review and Meta-Analysis of Malaria Test Positivity Outcomes and Programme Interventions in Low Transmission Settings in Southern Africa, 2000–2021
Source: Int J Environ Res Public Health. 2022 Jun 1;19(11):6776. doi: 10.3390/ijerph19116776 (PMC9180605; doi:10.3390/ijerph19116776)
Supplement: Supplementary file 1 [file ijerph-19-06776-s001.zip › ijerph-1698967-supplementary.pdf]

# SUPPLEMENTARY

## A Systematic Review and Meta-Analysis of Malaria Test Positivity Outcomes and Programme Interventions in low transmission settings in Southern Africa, 2000–2021

**Preferred Reporting Items for Systematic reviews and Meta-Analyses extension for Scoping Reviews (PRISMA-ScR) Checklist**

| SECTION | ITEM | PRISMA-ScR CHECKLIST ITEM | REPORTED ON PAGE # |
|---|---|---|---|
| **TITLE** | | | |
| Title | 1 | Identify the report as a scoping review. | 1 |
| **ABSTRACT** | | | |
| Structured summary | 2 | Provide a structured summary that includes(as applicable): background, objectives, eligibility criteria, sources of evidence, charting methods, results, and conclusions that relate to the review questions and objectives. | 1 |
| **INTRODUCTION** | | | |
| Rationale | 3 | Describe the rationale for the review in the context of what is already known. Explain why the review questions/objectives lend themselves to a scoping review approach. | 2-3 |
| Objectives | 4 | Provide an explicit statement of the questions and objectives being addressed with reference to their key elements (e.g., population or participants, concepts, and context) or other relevant key elements used to conceptualize the review questions and/or objectives. | 2-3 |
| **METHODS** | | | |
| Protocol and registration | 5 | Indicate whether a review protocol exists; state if and where it can be accessed (e.g., a Web address); and if available, provide registration information, including the registration number. | 4 |
| Eligibility criteria | 6 | Specify characteristics of the sources of evidence used as eligibility criteria (e.g., years considered, language, and publication status), and provide a rationale. | 5 |
| Information sources* | 7 | Describe all information sourcesin the search (e.g., databases with dates of coverage and contact with authors to identify additional sources), as well as the date the most recent search was executed. | 5 |

| SECTION | ITEM | PRISMA-ScR CHECKLIST ITEM | REPORTED ON PAGE # |
|---|---|---|---|
| Search | 8 | Present the full electronic search strategy for at least 1 database, including any limits used, such that it could be repeated. | 5 |
| Selection of sources of evidence | 9 | State the process for selecting sources of evidence (i.e., screening and eligibility) included in the scoping review. | 5 |
| Data charting process | 10 | Describe the methods of charting data from the included sources of evidence (e.g., calibrated forms or forms that have been tested by the team before their use, and whether data charting was done independently or in duplicate) and any processes for obtaining and confirming data from investigators. | 5 |
| Data items | 11 | List and define all variables for which data were sought and any assumptions and simplifications made. | 5 |
| Critical appraisal of individual sources of evidence | 12 | If done, provide a rationale for conducting a critical appraisal of included sources of evidence; describe the methods used and how this information was used in any data synthesis (if appropriate). | 5 |
| Synthesis of results | 13 | Describe the methods of handling and summarizing the data that were charted. | 6 |

| RESULTS | | | |
|---|---|---|---|
| Selection of sources of evidence | 14 | Give numbers of sources of evidence screened, assessed for eligibility, and included in the review, with reasons for exclusions at each stage, ideally using a flow diagram. | 6 |
| Characteristics of sources of evidence | 15 | For each source of evidence, present characteristics for which data were charted and provide the citations. | Click here to enter text. |
| Critical appraisal within sources of evidence | 16 | If done, present data on critical appraisal of included sources of evidence (see item 12). | Click here to enter text. |
| Results of individual sources of evidence | 17 | For each included source of evidence, present the relevant data that were charted that relate to the review questions and objectives. | 6-12 |
| Synthesis of results | 18 | Summarize and/or present the charting results as they relate to the review questions and objectives. | 6-12 |
| **DISCUSSION** | | | |
| Summary of evidence | 19 | Summarize the main results (including an overview of concepts, themes, and types of evidence available), link to the review questions and objectives, and consider the relevance to key groups. | 12-15 |
| Limitations | 20 | Discuss the limitations of the scoping review process. | 15 |
| Conclusions | 21 | Provide a general interpretation of the results with respect to the review questions and objectives, as well as potential implications and/or next steps. | 15 |
| **FUNDING** | | | |
| Funding | 22 | Describe sources of funding for the included sources of evidence, as well as sources of funding for the scoping review. Describe the role of the funders of the scoping review. | 16 |

*From:*Tricco AC, Lillie E, Zarin W, O'Brien KK, Colquhoun H, Levac D, et al. PRISMA Extension for Scoping Reviews (PRISMA-ScR): Checklist and Explanation. Ann Intern Med. ;169:467–473. doi: 10.7326/M18-0850

## SectionS2

### Search strategy – as was primarily used for PubMed search and applied in the other electronic databases.

((malaria programs) AND (low transmission settings)) AND (southern africa) Filters: Clinical Trial, Randomized Controlled Trial, from 2000 – 2022

((malaria programs) AND (low transmission settings)) AND (southern africa) Filters: Clinical Trial, Randomized Controlled Trial, from 2000 – 2022

(MALARIA TEST RESULTS) AND (SOUTHERN AFRICA) Filters: Clinical Trial, Randomized Controlled Trial, from 2000 – 2022

(MALARIA TEST AND TREAT) AND (SOUTHERN AFRICA) Filters: Clinical Trial, Randomized Controlled Trial, from 2000 – 2022

((MALARIA TEST POSITIVITY) AND (LOW TRANSMISSION SETTING)) OR (SOUTHERN AFRICA) Filters: Clinical Trial, Randomized Controlled Trial, from 2000 – 2022

((MALARIA TEST POSITIVE) AND (LOW TRANSMISSION SETTING)) AND (ZAMBIA) Filters: Clinical Trial, Randomized Controlled Trial

REACTIVE ACTIVE CASE DETECTION AND ESWATINI Filters: Clinical Trial, Randomized Controlled Trial

(REACTIVE ACTIVE CASE DETECTION) AND (ZAMBIA) Filters: Clinical Trial, Randomized Controlled Trial

((MALARIA) AND (APPROACH TO ELIMINATION)) AND (ZAMBIA) Filters: Clinical Trial, Randomized Controlled Trial

((MALARIA) AND (APPROACH TO ELIMINATION)) AND (SOUTH AFRICA) Filters: Clinical Trial, Randomized Controlled Trial

((MALARIA) AND (APPROACH TO ELIMINATION)) AND (NAMIBIA) Filters: Clinical Trial, Randomized Controlled Trial

## SectionS3

### MIXED METHODS APPRAISAL TOOL (MMAT)VERSION 2018

The MMAT is a critical appraisal tool that is designed for the appraisal stage of systematic mixed studies reviews, i.e., reviews that include qualitative, quantitative and mixed methods studies. It permits to appraise the methodological quality of five categories to studies: qualitative research, randomized controlled trials, non-randomized studies, quantitative descriptive studies, and mixed methods studies. Each of the question outcomes is remarked as ‘YES’, ‘NO’ or ‘CAN’T SAY’ with each positive weighted as 20% or represented as a star

**1. Qualitative**

1.1. Is the qualitative approach appropriate to answer the research question?
1.2. Are the qualitative data collection methods adequate to address the research question?
1.3. Are the findings adequately derived from the data?
1.4. Is the interpretation of results sufficiently substantiated by data?
1.5. Is there coherence between qualitative data sources, collection, analysis and interpretation?

**2. Quantitative randomized controlled trials**
2.1. Is randomization appropriately performed?
2.2. Are the groups comparable at baseline?
2.3. Are there complete outcome data?
2.4. Are outcome assessors blinded to the intervention provided?
2.5 Did the participants adhere to the assigned intervention?

**3. Quantitative non-randomized**
3.1. Are the participants representatives of the target population?
3.2. Are measurements appropriate regarding both the outcome and intervention (or exposure)?
3.3. Are there complete outcome data?
3.4. Are the confounders accounted for in the design and analysis?
3.5. During the study period, is the intervention administered (or exposure occurred) as intended?

**4. Quantitative descriptive**
4.1. Is the sampling strategy relevant to address the research question?
4.2. Is the sample representative of the target population?
4.3. Are the measurements appropriate?
4.4. Is the risk of non-response bias low?
4.5. Is the statistical analysis appropriate to answer the research question?

**5. Mixed methods**

5.1. Is there an adequate rationale for using a mixed methods design to address the research question?
5.2. Are the different components of the study effectively integrated to answer the research question?
5.3. Are the outputs of the integration of qualitative and quantitative components adequately interpreted?

5.4. Are divergences and inconsistencies between quantitative and qualitative results adequately addressed?
5.5. Do the different components of the study adhere to the quality criteria of each tradition of the methods involved?

Reference

Hong, Q. N., Fàbregues, S., Bartlett, G., Boardman, F., Cargo, M., Dagenais, P., . . . Pluye, P. (2018). The Mixed Methods Appraisal Tool (MMAT) version 2018 for information professionals and researchers. *Education for Information, **34***, 285-291. doi:10.3233/EFI-180221

**Supplementary Table S1**

# Characteristics of included studies according to the Mixed Method Appraisal Tool (MMAT)

| | AUTHOR | YEAR | COUNTRY | STUDY TYPE | Outcome | MMAT score |
|---|---|---|---|---|---|---|
| 1 | Bhondoekhan et al | 2020 | ZAMBIA | Quantitative descriptive | **** | 80% |
| 2 | Pringle et al | 2019 | Zambia | Quantitative descriptive | ***** | 100% |
| 3 | Tambo et al | 2018 | Namibia | Quantitative RCT | ***** | 100% |
| 4 | Smith et al | 2017 | Namibia | Quantitative RCT | ***** | 100% |
| 5 | Hsiang et al | 2019 | Eswatini | Quantitative descriptive | ***** | 100% |
| 6 | Deutsch-Feldman et al | 2018 | Zambia | Quantitative descriptive | **** | 80% |
| 7 | Larsen et al | 2017 | Zambia | Quantitative non RCT | ***** | 100% |
| 8 | Sturrock et al | 2013 | Eswatini | Quantitative descriptive | ***** | 100% |
| 9 | Pinchoff et al | 2015 | Zambia | Quantitative RCT | **** | 80% |
| 10 | Larsen et al | 2015 | Zambia | Quantitative descriptive | ***** | 100% |
| 11 | Hsiang et al | 2020 | Namibia | Quantitative RCT | ***** | 100% |
| 12 | Tejedor-Garavot et al | 2017 | Eswatini | Quantitative descriptive | ***** | 100% |
| 13 | Mulenga et al | 2006 | Zambia | Quantitative RCT | ***** | 100% |
| 14 | Vilakati et al | 2021 | Eswatini | Quantitative RCT | ***** | 100% |

| | | | | | | |
|---|---|---|---|---|---|---|
| 15 | Eisele et al | 2016 | Zambia | Quantitative RCT | ***** | 100% |
| 16 | Hamer et al | 2012 | Zambia | Quantitative RCT | ***** | 100% |
| 17 | Chanda et al | 2006 | Zambia | Quantitative descriptive | **** | 80% |
| 18 | Finn et al | 2020 | Zambia | Quantitative descriptive | **** | 80% |

*Each positive respond is marked YES and weighted 20% and this can also be represented as star (*). 72% of the articles met all the criteria showing that they are of the high quality necessary for inclusion in this review; none of the articles involved had a quality estimate of less than 80% on the MMAT scale.*

**SUPPLEMENTARY TABLE S2**

**POINTS OF NOTE IN THE SYSTEMATIC REVIEW**

| AUTHOR/YEAR | COUNTRY | NO SCREENED | TOTAL TESTED POSITIVE | DIAGNOSTIC METHOD | STUDY TYPE | FINDING |
|---|---|---|---|---|---|---|
| Mulenga et al., 2006 | Zambia | 1048 | 255 | Microscopy/PCR | Double blind randomised | A total of 128 children with a packed cell volume of 9% and P. falciparum parasitaemia received treatment with AP and 127 treatment with SP. Treatment failure occurred in 28 children (22%) who received SP and in 10 (8%) who received AP (OR: 3.34, 95% CI: 1.54, 7.21). |
| Chanda et al., 2006 | Zambia | 953 | 111 | Microscopy | Prospective | 91/111 children enrolled in the study, were successfully followed up. Artemether lumefantrine was found to produce significant gametocyte reduction. The Adequate Clinical and Parasitological Response was found to be 100% (95% CI 96.0;100) |
| Hammer et al., 2012 | Zambia | 975 | 270 | RDT | Cluster randomised | During the 12-month study, the CHWs evaluated 1017 children with fever and/or fast/difficult breathing and performed 975 RDTs. Malaria and/or pneumonia were appropriately classified 94–100% of the time. Treatment based on disease classification was correct in 94–100% of episodes. |
| Eisele et al., 2016 | Zambia | 5018 | 1097 | RDT | Cluster randomised | All end points significantly decreased after intervention, irrespective of treatment group. Parasite prevalence from 7.71% at baseline to 0.54% after MDA in lower-transmission areas, resulting in an 87% reduction compared with control (adjusted odds ratio, 0.13; 95% confidence interval, .02–.92; P = .04). No difference between treatment groups was observed in areas of high transmission. No significant impact of focal MDA was observed for any end point |
| Sturrock et al., 2013 | Eswatini | 3671 | 74 | RDT | Cohort study | Over the study period, 250 cases triggered RACD, which identified a further 74 cases, showing the value of RACD over |

| | | | | | | |
|---|---|---|---|---|---|---|
| | | | | | | passive surveillance alone. Results suggest that the odds of detecting a case within the household of the index case were significantly higher than in neighbouring households (odds ratio (OR) 13, 95% CI 3.1–54.4). |
| Pinchoff et al., 2015 | Zambia | 1621 | 735 | RDT | Cohort study | A total of 426 index households were enrolled, with 1,621 household contacts (45% RDT positive). Two space–time clusters were identified in the rainy season, with ten times and six times higher risk than expected. Significantly increased spatial clustering of index households was found in the rainy season as compared to the dry season (based on K-function methodology). However, no seasonal difference in mapped spatial intensity of index households was identified. |
| Larsen et al., 2015 | Zambia | 143,295 | 22,201 | RDT | Descriptive cross sectional | RACD was used for targeting malaria interventions, and was instrumental for guid ing focal indoor residual spraying in Lusaka during the 2014/2015 spray season. Variations to maximize impact of the current RCD protocol that were considered, including the use of anti-malarials with a longer lasting, post-treatment prophylaxis. |
| Tejedor-Garavot et al., 2017 | Eswatini | 9859 | 105 | RDT | Ecological study | Of 1517 confrmed cases identifed through passive surveillance, 67% reported travel history. A large proportion of positive cases reported domestic or international travel history (65%) compared to negative cases (10%). The primary risk factor for malaria infection in Swaziland was shown to be travel, more specifcally international travel to Mozambique by 25- to 44-year old males, who spent on average 28 nights away. Additionally, paths of transmission, important border crossings and means of transport were identifed. |
| Larsen et al., 2017 | Zambia | 14,409 | 1200 | RDT | Retrospective cohort | Location was a more powerful predictor of finding malaria infections during case investigations than the demographics of the incident case. After accounting for environmental characteristics, no demographics around the incident case were associated with finding malaria infections during case investigations. Various time-invariant measures of the |

| | | | | | | |
|---|---|---|---|---|---|---|
| | | | | | | environment, such as median enhanced vegetation index, the topographic position index, the con- vergence index, and the topographical wetness index, were all associated as expected with increased probability of finding a malaria infection during case investigations. |
| Smith et al., 2017 | Namibia | 3151 | 89 | RDT, LAMP | Prospective case control | Prevalence of Plasmodium falciparum infection by LAMP was 3.4%, 1.4% and 0.4% in index-case households, neighbors of index case households and control households respectively; adjusted odds ratio 6.1 [95%CI 1.9–19.5] comparing case households versus control households. Using data from Engela, neighbors of cases had higher odds of infection [adjusted OR 5.0 95%CI 1.3–18.9] compared to control households. All infections identified by RDTs were afebrile and RDTs identified only a small proportion of infections in case (n = 7; 17%) and control (0%) neighborhoods. |
| Tambo et al., 2018 | Namibia | 2642 | 47 | RDT LAMP qPCR | Prospective case control | Some 3151 individuals were tested by RDT, LAMP and nPCR. Sensitivity of RDTs and LAMP were 9.30 and 95.50%, respectively, and specifcities were 99.27 and 99.92%, respectively, compared to nPCR. LAMP carried out on collected RDTs showed a sensitivity and specifcity of 95.35 and 99.85% compared to nPCR carried out on DBS. There were 2 RDT samples that were negative by LAMP but the corresponding DBS samples were positive by PCR. |
| Deutsch-Feldman et al., 2018 | Zambia | 3016 | 73 | RDT, qPCR | Prospective observational | From January 2015 through March 2016, 145 index cases were identified at health centers and health posts. A total of 3,333 individuals residing in 525 households were screened. Excluding index cases, the parasite prevalence was 1.1% by RDT (33 positives of 3,016 participants) and 2.4% by qPCR (73 positives of 3,016 participants). Of the qPCR-positive cases, 62% of 73 individuals tested negative by RDT. Approximately half of the infected individuals resided within the index case household (58% of RDT-positive individuals and 48% of qPCR-positive individuals). |

| | | | | | | |
|---|---|---|---|---|---|---|
| Hsiang et al., 2020 | Eswatini | 10890 | 180 | RDT, LAMP | Prospective | Among 377 RACD events, 10 890 participants residing within 500 m of index cases were tested. Compared to RDT, LAMP provided a 3-fold and 2.3-fold higher yield to detect infections (1.7% vs 0.6%) and hotspots (29.7% vs 12.7%), respectively. Hotspot detection improved with ≥80% target population coverage and response times within 7 days. Proximity to the index case was associated with a dose-dependent increased infection risk (up to 4-fold). Individual-, index case–, and other RACD-level factors were considered but the simple approach of restricting RACD to a 200-m radius maximized yield and efficiency |
| Finni et al., 2020 | Zambia | 597,631 | 30,898 | RDT microscopy | Cluster randomised | Adherence information was reported for 181,534 of 336,821 DHAp (53.9%) treatments administered during four rounds of MDA/fMDA, of which 153,197 (84.4%) reported completing the full course of DHAp. The proportion of participants fully adhering to the treatment regimen differed by MDA modality (MDA versus fMDA), RDT status, and whether the first dose was observed by those administering treatments. Among a subset of participants receiving DHAp and selected for longitudinal follow-up, 58 were positive for asexual-stage P. falciparum infection by microscopy at baseline. |
| Hsiang et al., 2020 | Namibia | 4,701 | 178 | RDT, LAMP | Cluster randomised | s Between Jan 1, 2017, and Dec 31, 2017, 55 enumeration area clusters had 1118 eligible index cases that led to 342 interventions covering 8948 individuals. The cumulative incidence of locally acquired malaria was 30·8 per 1000 person-years (95% CI 12·8–48·7) in the clusters that received rfMDA versus 38·3 per 1000 person-years (23·0–53·6) in the clusters that received RACD; 30·2 per 1000 person-years (15·0–45·5) in the clusters that received RAVC versus 38·9 per 1000 person-years (20·7–57·1) in the clusters that did not receive RAVC; and 25·0 per 1000 person-years (5·2–44·7) in the clusters that received rfMDA plus RAVC versus 41·4 per 1000 person-years (21·5–61·2) in the clusters that received RACD only. |

| | | | | | | |
|---|---|---|---|---|---|---|
| Bhondoekhan et al., 2020 | Zambia | 4170 | 153 | RDT &qPCR | Cross sectional | The parasite prevalence in secondary (non-index case) households was 0.7% by RDT and 1.8% by qPCR. Overall, 8.5% (n=45) of secondary households had at least one resident with parasitaemia by qPCR or RDT. The risk of a secondary household having a parasitaemic resident was signifcantly increased in proximity to higher order streams and marginally with increasing distance from index households. The adjusted OR for proximity to third- and ffth-order streams were 2.97 (95% CI 1.04–8.42) and 2.30 (95% CI 1.04–5.09), respectively, and that for distance to index households for each 50 m was 1.24 (95% CI 0.98–1.58). |
| Vilakati et al., 2021 | Eswatini | 1455 | 5 | RDT/LAMP | Cluster randomised | From September 2015 to August 2017, 222 index cases from 47 clusters triggered 46 RACD events and 64 rfMDA events. RACD and rfMDA were delivered to 1455 and 1776 individuals, respectively. Index case coverage was 69.5% and 62.4% for RACD and rfMDA, respectively. Adherence to DP was 98.7%. For rfMDA versus RACD, cumulative incidences (per 1000 person-years) of all malaria were 2.11 (95% CI 1.73 to 2.59) and 1.97 (95% CI 1.57 to 2.47), respectively; and of locally acquired malaria, they were 1.29 (95% CI 1.00 to 1.67) and 0.97 (95% CI 0.71 to 1.34), respectively. |

**Supplementary Table S3**

**OUTCOME CONCLUSIONS FROM STUDIES USED IN THE SYSTEMATIC REVIEW**

| AUTHOR/YEAR | CONCLUSION |
|---|---|
| Mulenga et al., 2006 | In an area with a modest level of sulphadoxine–pyrimethamine resistance atovaquone–proguanil proved to be more effective than sulphadoxine–pyrimethamine in the treatment of malarial anaemia.<br>The availability of a more effective antimalaria reduces the need for blood transfusion in children with malarial anaemia |
| Chanda et al., 2006 | Artemether-lumefantrine was effective in treating uncomplicated malaria in children weighing less than 10 kg. |
| Hammer et al., 2012 | With adequate training and supervision, community health workers are capable of providing integrated management of malaria and pneumonia with quality output. |
| Eisele et al., 2016 | In low-transmission areas, when dihydroartemisinin–piperaquine was applied in two rounds of mass drug administration there was rapidly reduced infection prevalence, infection incidence, and confirmed case incidence rates. |
| Sturrock et al., 2013 | Reactive case detection would be more effective by achieving high coverage amongst individuals located near index cases and in areas where spraying has not been conducted. |
| Pinchoff et al., 2015 | Clinic-based interventions will miss asymptomatic, non-care seeking infections located farther from the road.<br>RACD may identify additional infections missed at the clinic |
| Larsen et al., 2015 | Reactive case detection provides an additive to routine surveillance system leading to better characterization of malaria burden and guide for intervention. |
| Tejedor-Garavot et al., 2017 | As countries drive towards malaria elimination collaboration between neighbouring countries is needed to tackle the importation of malaria. |
| Larsen et al., 2017 | Targeting the locations that are highly at risk of malaria transmission than the demographics of the incident case is of importance in elimination settings |
| Smith et al., 2017 | Malaria infections cluster around passively detected cases majority of which are asymptomatic and of densities below the limit of detection of current RDTs.<br>Reactive case detection using standard RDTs are unlikely to detect enough malaria infections to dramatically reduce transmission.<br>In low transmission more sensitive field diagnostics or forms of focal presumptive treatment should be tested as strategies to reduce malaria transmission. |
| Tambo et al., 2018 | LAMP had the equivalent performance as nested Polymerase Chain Reaction for the identification of Plasmodium falciparum infection. LAMP is particularly useful in elimination settings where high sensitivity and ease of operation are important. |
| Deutsch-Feldman et al., 2018 | The efficiency of reactive test-and-treat in the study is notably decreased due to low sensitivity of the RDT and the high proportion of secondary cases within the index case household.<br>Reactive focal drug administration in index case households would be a more efficient approach to treating infected individuals associated with a symptomatic case |
| Hsiang et al., 2020 | Conducting of RACD should be done using more sensitive diagnostics and clear context-specific operational parameters. |

| | |
|---|---|
| Finni et al., 2020 | Dihydroartemisinin–piperaquine provided a 100% short-term clearance of asexual P. falciparum parasite infections.<br>Dihydroartemisinin–piperaquine are effective when administered through mass drug administration or focal mass drug administration rounds for clearing asexual P. falciparum parasite infections in spite of its recorded outside of Africa |
| Hsiang et al., 2020 | In a low malaria-endemic setting, reactive focal mass drug administration and reactive focal vector control, implemented alone and in combination, reduced malaria transmission and should be considered as alternatives to RACD for elimination of malaria |
| Bhondoekhan et al., 2020 | The efficiency of reactive case detection can be improved when environmental factors are alongside considered as screening strategy. |
| Vilakati et al., 2021 | Reactive focal mass drug administration using dihydroartemisinin–piperaquine in very low transmission setting was safe but did not lower malaria incidence cases when compared with reactive case detection which made use of artemether–lumefantrine. |

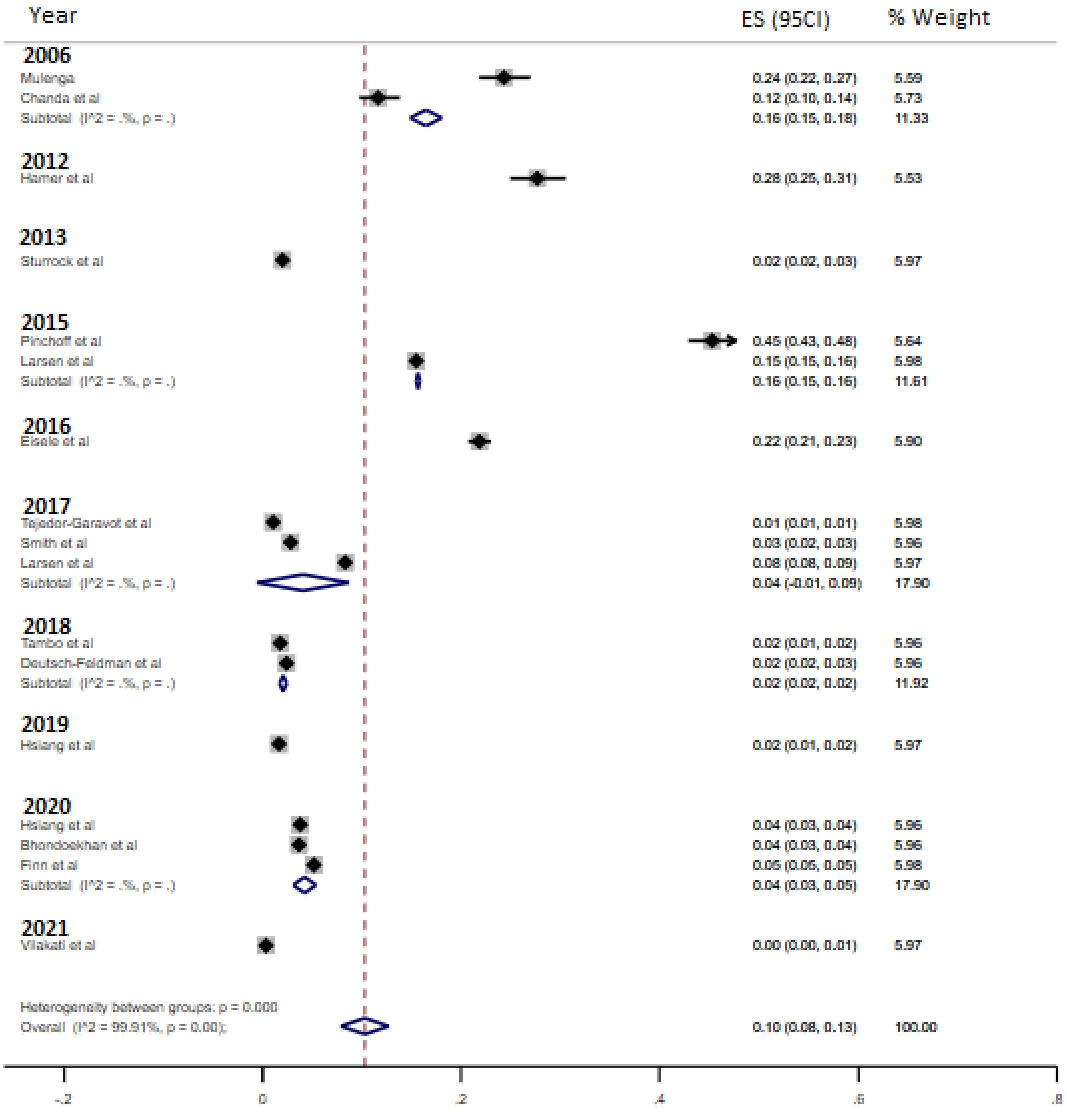

**Figure S1:**Forest plot depicting subgroup analysis of malaria test positivity incidence based on the year of publication
